# Detection of Organophosphorus, Pyrethroid, and Carbamate Pesticides in Tomato Peels: A Spectroscopic Study

**DOI:** 10.3390/foods14142543

**Published:** 2025-07-21

**Authors:** Acela López-Benítez, Alfredo Guevara-Lara, Diana Palma-Ramírez, Karen A. Neri-Espinoza, Rebeca Silva-Rodrigo, José A. Andraca-Adame

**Affiliations:** 1 Departamento de Nanomateriales, Unidad Profesional Interdisciplinaria de Ingeniería Campus Hidalgo (UPIIH), Instituto Politécnico Nacional (IPN), San Agustín Tlaxiaca 42162, Hidalgo, Mexico; dpalmar@ipn.mx (D.P.-R.); kneri@ipn.mx (K.A.N.-E.); 2 Área Académica de Química, Instituto de Ciencias Básicas e Ingeniería (ICBI), Universidad Autónoma del Estado de Hidalgo (UAEH), Mineral de la Reforma 42184, Hidalgo, Mexico; guevaraa@uaeh.edu.mx; 3 Centro de Investigación en Petroquímica, Tecnológico Nacional de México, Instituto Tecnológico de Ciudad Madero, Ciudad Madero 89600, Tamaulipas, Mexico; rebeca.sr@cdmadero.tecnm.mx

**Keywords:** pesticides, methomyl, dichlorvos, tomato, FT-IR, Raman spectroscopy

## Abstract

Tomatoes are among the most widely consumed and economically significant fruits in the world. However, the extensive use of pesticides in their cultivation has led to the contamination of the peels, posing potential health risks to consumers. As one of the top global producers, consumers, and exporters of tomatoes, Mexico requires rapid, non-destructive, and real-time methods for pesticide monitoring. In this study, a detailed characterization of six pesticides using Raman and Fourier Transform Infrared (FT-IR) spectroscopies was carried out to identify their characteristic vibrational modes. The pesticides examined included different chemical classes commonly used in tomato cultivation: organophosphorus (dichlorvos and methamidophos), pyrethroids (lambda-cyhalothrin and cypermethrin), and carbamates (methomyl and benomyl). Tomato peel samples were examined both before and after pesticide application. Prior to treatment, the peel exhibited a well-organized polygonal structure and showed the presence of carotenoid compounds. After pesticide application, no visible structural damage was observed; however, distinct vibrational bands enabled the detection of each pesticide. Organophosphorus pesticides could be identified through vibrational bands associated with P-O and C-S bonds. Pyrethroid detection was facilitated by benzene ring breathing modes and C=C stretching vibrations, while carbamates were identified through C-N stretching contributions. Phytotoxicity testing in the presence of pesticides indicates no significant damage during the germination of tomatoes.

## 1. Introduction

Tomato (*Solanum lycopersicum*) is one of the most widely cultivated crops worldwide [1]. It is also among the most versatile and commonly consumed foods, valued for its rich nutritional content, including antioxidants, minerals, vitamins, and carotenoids [2,3]. According to the Food and Agriculture Organization (FAO) of the United Nations, global tomato production reached 192 million tonnes in 2023 [4]. Despite their economic and nutritional significance, tomato plants are particularly susceptible to pest infestations, which has led to the widespread use of pesticides to protect crop yield and quality [5]. Consequently, tomatoes are listed among the top ten fruits and vegetables most contaminated with pesticide residues [6]. These chemicals can remain on the surface of the fruit or penetrate the tissue, posing potential risks to consumers [1].

Given the global scale of tomato consumption, a wide range of pesticide classes has been used, including organochlorines, organophosphorus, pyrethroids, and carbamate compounds [1,7,8,9]. Pesticide exposure has been correlated with various acute and chronic health conditions, including asthma, neurodegenerative disorders, diabetes, leukemia and Parkinson’s disease [10,11]. Due to their toxicity, the use of many of these pesticides has been restricted or banned in several countries [12].

Mexico is one of the leading producers and exporters of tomatoes globally, supplying both domestic and international markets [13,14]. Consequently, the implementation of reliable, accessible, and efficient methods for detecting pesticide residues is crucial to meet export quality standards. However, the analysis of pesticide residues in biological samples remains a persistent challenge due to the complexity and variability of sample matrices, as well as the typically low concentrations of residues [15]. Several analytical techniques have been employed to detect pesticide residues on the surfaces of vegetables and fruits, including liquid and gas chromatography [16,17], gas chromatography-mass spectrometry (GC-MS) [18], high-performance liquid chromatography (HPLC) [19], biosensors [20], and immunoassays [21]. While these methods offer high precision and sensitivity, they usually involve complex sample preparation, long processing times, and costly instrumentation, making them less suitable for field use or routine monitoring [22].

Surface-enhanced Raman spectroscopy (SERS) has been widely used for pesticide detection in food samples [23,24]. This technique enables both the detection and quantification of a wide range of pesticide residues, even at trace levels [25]. However, SERS requires specialized instrumentation and the use of nanostructured materials (typically based on gold, silver, or modified substrates) to perform the analysis effectively [26,27]. In contrast, conventional Raman spectroscopy is a well-established analytical method in food safety and quality analysis. It enables molecular identification through characteristic vibrational fingerprints [28]. Raman spectroscopy is emerging as a promising tool for pesticide detection due to its advantages, including portability, real-time monitoring, rapid and non-destructive analysis, and minimal or no sample preparation [22,29]. These features make it particularly suitable for field applications, agricultural market quality control, and on-site food safety monitoring. When combined with FT-IR spectroscopy, Raman analysis provides complementary molecular information, enhancing confidence in the detection and characterization of pesticide residues in complex food matrices.

In this study, we employed conventional Raman spectroscopy and Fourier Transform Infrared (FT-IR) analysis to detect organophosphorus (dichlorvos and methamidophos), pyrethroid (lambda-cyhalothrin and cypermethrin), and carbamate (methomyl and benomyl) pesticides in tomato peel samples. The tomatoes were sourced from Hidalgo, a state in central Mexico. The selected pesticides are frequently used in tomato cultivation [30,31,32], and their inclusion was based on their continued widespread application in this region. Notably, detection was performed using portable Raman equipment without any sample pretreatment, offering a rapid, practical, and real-time analysis approach. Although Raman spectroscopy has previously been utilized for pesticide detection, most studies have employed spectrometers equipped with 532 nm or 785 nm lasers [33,34], which often produce weak signals due to fluorescence interference. In contrast, using a Raman spectrometer equipped with a 1064 nm laser significantly reduces fluorescence and improves spectral quality in biological samples [35]. However, few studies have employed this type of equipment for pesticide detection [36]. The aim of this work is to detect organophosphorus, pyrethroid, and carbamate pesticides in tomato peel samples using a 1064 nm Raman spectrometer without the need for sample pretreatment. FT-IR analysis was also conducted to validate and complement the identification of pesticide residues. The spectroscopic results are provided to identify the characteristic vibrational modes of each pesticide and to provide a basis for their detection in food products. Finally, a phytotoxicity test was conducted on tomato plants germinated in the presence of each pesticide to assess their specific effects on plant development.

## 2. Materials and Methods

### 2.1. Chemical Materials and Reagents

Pesticide reagents of dichlorvos (technical grade, 98%), lambda-cyhalothrin (technical grade, 95%), cypermethrin (technical grade, 94%), benomyl (technical grade, 95%), and methomyl (technical grade, 98%) were purchased from Shanghai Lavaur Chemical Co., Ltd. (Shanghai, China). Methamidophos (technical grade, 73%) was obtained from Green Mountain Co., Ltd. (Taiwan, China). Methanol (ACS reagent ≥ 99.8%) was supplied by J.T. Baker (Phillipsburg, NJ, USA).

### 2.2. Preparation of the Tomato Peel Samples Treated with Pesticides

Fresh tomatoes were obtained from a local market in Pachuca City, Hidalgo, Mexico, in September 2024. The experimental procedure was based on the method reported by Ma et al. [37]. Tomatoes at the red-ripening stage were selected and thoroughly washed with methanol and deionized water to remove surface impurities. Six tomato peel samples were prepared by slicing the tomatoes into pieces approximately 2 cm × 1 cm in size and 1 mm in thickness. Each peel sample was then placed on a glass slide. Subsequently, 0.01 mL of each pesticide solution (50% *v/v* in deionized water) of dichlorvos, methamidophos, lambda-cyhalothrin, cypermethrin, methomyl, and benomyl was applied to individual tomato peel samples. Prior to analysis, the samples were allowed to stand for four hours to ensure surface stabilization. An additional sample, untreated with pesticides, was included as a control.

### 2.3. Phytotoxicity Test

Phytotoxicity was analyzed in accordance with the OECD 208 test, which evaluates the effects of exposure to pesticide substances applied into soil on seedling emergence and early growth of higher plants.

The soil used was dried at 50 °C for 24 h in an oven. Soil particles were then sieved through a 2 mm mesh to homogenize the sample and remove coarse particles. Soil with an organic carbon content of up to 1.5% by weight (approximately 3% organic matter) was employed.

**Figure 1:**
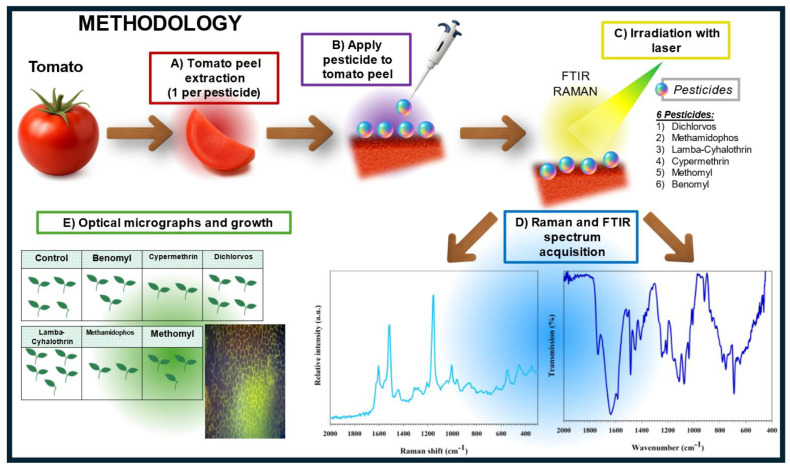
Tomato sample preparation and experimental procedure used for pesticide application and analysis.

Pesticide solutions were prepared in advance using deionized water (1000 mg/L). Five tomato seeds were placed in 20 g of soil in each compartment of a twelve-section tray. Then, 2 mL of the prepared pesticide solution was added to each soil sample using a Pasteur pipette. All samples of pesticides in the soil were tested in triplicate to ensure repeatability, i.e., three compartments of the trays were employed for each pesticide. The same procedure was conducted considering only the soil sample (control), with three compartments for the control (triplicate). The trays were placed in a germination chamber under a photoperiod of 16 h of ultraviolet light and 8 h of darkness. Each compartment was irrigated daily with 2 mL of deionized water. After 50% of the seeds in the control had germinated, they were monitored for 14 days. Temperature (25 °C), relative humidity (46%), carbon dioxide concentration (550 ppm), and light (1542 lux) were monitored. Additionally, light periods and irrigation were monitored daily to ensure adequate plant growth. Plants were also visually monitored daily. The percentage of seed emergence and the biomass of surviving plants were calculated, along with an analysis of any detrimental effects on different plant parts. In addition, fresh shoot weight, length, and width were analyzed for each plant. The results are the average of the samples in triplicate. The sample preparation process and experimental procedure are shown in Figure 1.

### 2.4. Characterization Techniques

Raman spectra were acquired using a portable BWTEK iRamanPlus spectrometer (B&W TEK, Newark, DE, USA) equipped with a 1064 nm excitation laser and an HQE-CCD detector. For each sample, an average of 20–40 scans were acquired. The laser intensity ranged from 50 to 70 mW. Spectral measurements were recorded in the 2000–450 cm^−1^ range, with a resolution of 3 cm^−1^. The instrument was also equipped with a microscope (20, 50, and 100×), which was used to obtain optical micrographs.

Infrared spectra were recorded using a PerkinElmer Frontier FT-IR spectrometer (Perkin Elmer, Inc. Waltham, MA, USA) equipped with an ATR accessory (Gladi; PIKE Technologies, Madison, WI, USA).

## 3. Results

### 3.1. Spectroscopic Characterization of Pesticides

#### 3.1.1. Organophosphorus Pesticides

**Figure 2:**
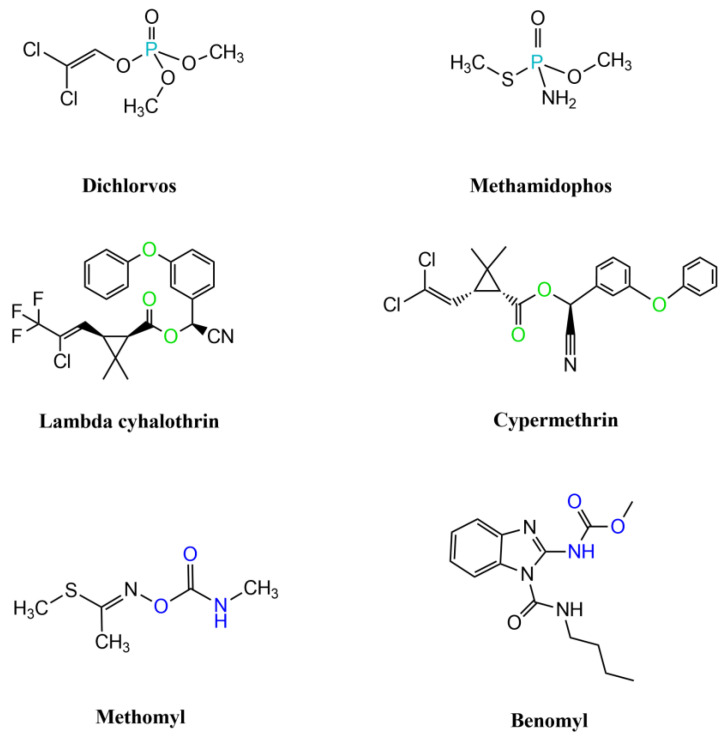
Organophosphorus, pyrethroid, and carbamate pesticides analyzed in this study.

Pesticide reagents were analyzed using Raman and FT-IR spectroscopy. The chemical structures of the pesticides analyzed are shown in Figure 2.

**Figure 3:**
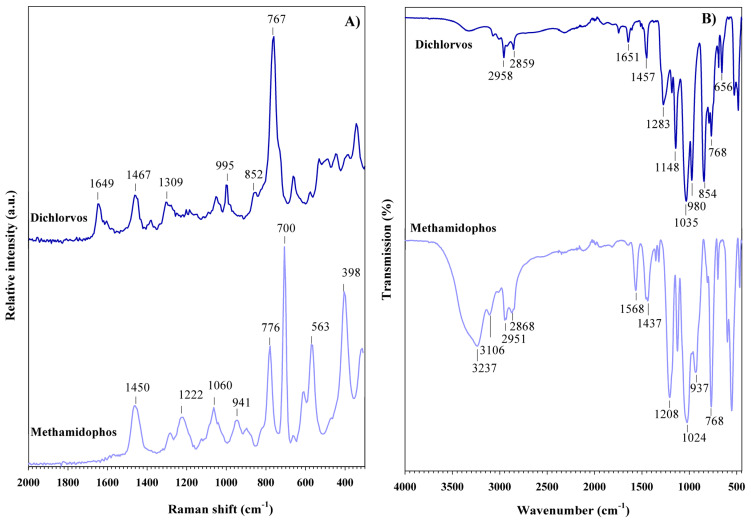
(**A**) Raman spectra and (**B**) FT-IR spectra of organophosphorus pesticides.

Figure 3 shows the Raman and FT-IR spectra of organophosphorus pesticide reagents. The Raman spectrum of dichlorvos (Figure 3A) exhibits an intense band at 767 cm^−1^, which is attributed to the P-O stretching vibration and commonly used to identify this pesticide. Bands at 852 and 995 cm^−1^ are assigned to the in-phase and out-of-phase P-O-C stretching vibrations, respectively [38]. Additional bands at 1309 and 1467 cm^−1^ are associated with the vibrations of the P=O and CH_3_ groups, respectively. Finally, the band at 1649 cm^−1^ is attributed to the C=C bond [39].

In the FT-IR spectrum of dichlorvos (Figure 3B), all previously mentioned vibrational features are observed, with slight shifts in wavenumber. Additional bands are also detected; in particular, those at 656, 1035, and 1148 cm^−1^ correspond to C-Cl, P-O-C, and C-O stretching vibrations, respectively [40,41]. The characteristic C-H stretching vibrations of the vinyl group are notably present between 2859 and 2958 cm^−1^ [42].

The Raman spectrum of methamidophos is also shown in Figure 3A. Two bands are observed at 398 and 563 cm^−1^, corresponding to N-H twisting and wagging vibrations, respectively. The most intense band of this pesticide is observed at 700 cm^−1^ and is attributed to the symmetric stretching vibration of the C-S bond. The band at 776 cm^−1^ is associated with both P-O stretching and N-H wagging modes [43]. The band at 941 cm^−1^ is assigned to overlapping ν(C-O) and ν(P-O) stretching vibrations. Additionally, bands at 1060, 1222, and 1450 cm^−1^ are attributed to CH_3_ rocking, PO_2_ stretching, and CH_3_ bending vibrations, respectively [44]. Compared to the Raman spectrum, the FT-IR spectrum of methamidophos (Figure 3B) shows these bands slightly shifted to lower wavenumbers. For instance, the PO_2_ stretching vibration appears at 1222 cm^−1^ in the Raman spectrum, while it is observed at 1208 cm^−1^ in the FT-IR spectrum. Further contributions at 1568, 2951, and 3237 cm^−1^ are assigned to δ(NH_2_), ν(CH_3_), and ν(NH_2_) vibrations, respectively [44]. The Raman and FT-IR bands, along with their corresponding vibrational mode assignments, of the organophosphorus pesticides discussed are summarized in Table 1.

#### 3.1.2. Pyrethroid Pesticides

**Figure 4:**
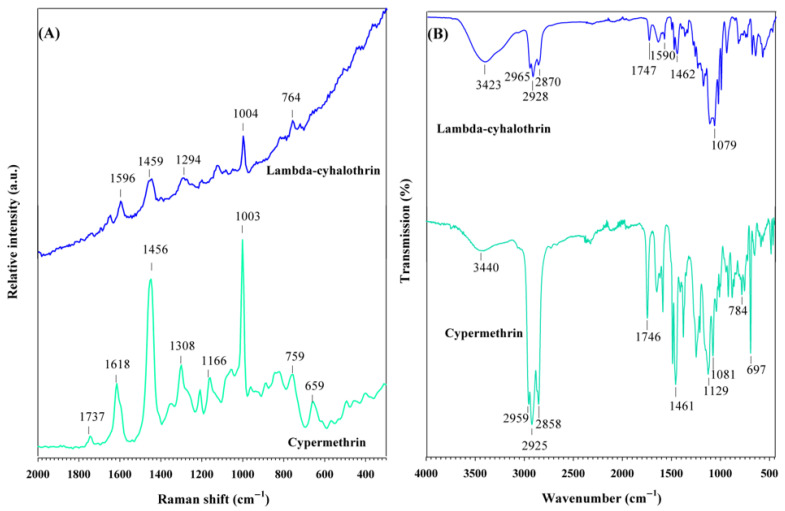
(**A**) Raman spectra and (**B**) FT-IR spectra of pyrethroid pesticides.

Pyrethroids were also characterized by Raman and FT-IR spectroscopies, as shown in Figure 4. Lambda-cyhalothrin exhibits five distinct Raman bands, consistent with those reported by Atanasov et al. [45]. The bands at 764 and 1004 cm^−1^ correspond to the in-plane deformation mode and the breathing mode of the benzene ring, respectively (Figure 4A). The band at 1294 cm^−1^ is attributed to the C-O stretching vibration, while the bands at 1459 and 1596 cm^−1^ are assigned to the C-H scissoring vibration of the cyclopropyl group and the stretching vibration of the benzene ring, respectively [45,46]. In the FT-IR spectrum of lambda-cyhalothrin, an intense band is observed at 1079 cm^−1^, corresponding to the C-O stretching vibration [47], (Figure 4B). The benzene ring stretching vibrations appear at 1462 and 1590 cm^−1^, while the C-H stretching vibrations are observed in the range of 2870–2965 cm^−1^ [48,49]. Finally, a broad band at 3423 cm^−1^ is associated with the O-H vibration of the solvent.

The Raman spectrum of cypermethrin (Figure 4A) shows bands of higher intensity compared to its homologue, lambda-cyhalothrin. Two bands are observed at 659 and 759 cm^−1^, corresponding to the in-plane deformation of the cyclopropyl group and the out-of-plane bending of the benzene ring, respectively. The most intense band in cypermethrin is observed at 1003 cm^−1^, attributed to the breathing vibration of the benzene ring. Additional bands at 1166 and 1308 cm^−1^ correspond to the C-H scissoring vibration and the skeletal vibration of the benzene ring, respectively. The strong band at 1456 cm^−1^ is associated with the asymmetric C-H scissoring vibration of the CH_3_ group. Finally, the bands at 1618 and 1737 cm^−1^ are assigned to the ν(C=C) stretching vibration of the vinyl group and the ν(C=O) stretching vibration of ester group, respectively [50]. In the FT-IR spectrum of cypermethrin, contributions from the C=C and C=O stretching vibrations are observed at 1461 and 1746 cm^−1^, respectively. C-H stretching vibrations are detected between 2858 and 2959 cm^−1^ [51], (Figure 4B). Table 1 summarizes the Raman and FT-IR results of pyrethroid pesticides.

#### 3.1.3. Carbamate Pesticides

**Figure 5:**
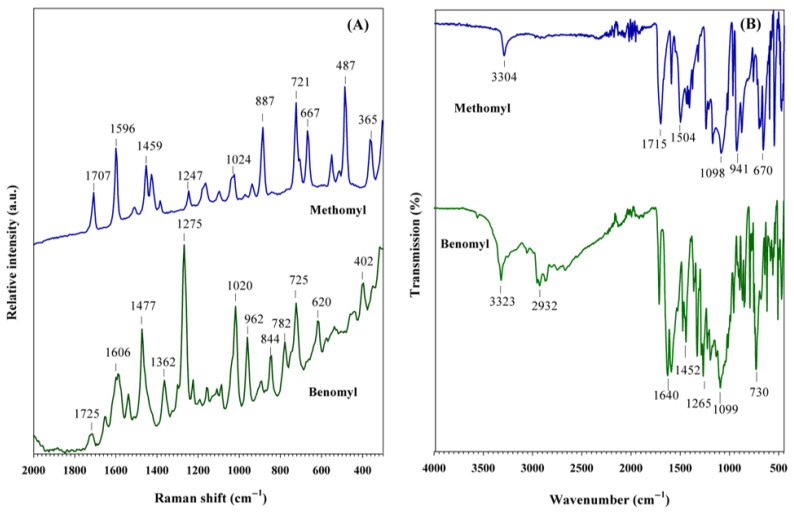
(**A**) Raman spectra and (**B**) FT-IR spectra of carbamate pesticides.

The Raman and FT-IR spectra of the carbamate pesticides are shown in Figure 5. Both solid samples exhibit several characteristic bands. Methomyl exhibits Raman bands at 365, 487, 667, and 721 cm^−1^, which are associated with NC_2_ bending, C=O rocking, and the latter two with S-CH_3_ stretching vibrations, respectively [52], (Figure 5A). Bands at 887, 1024, and 1247 cm^−1^ correspond to C-N stretching, N-CH_3_ rocking, and NC_2_ asymmetric stretching vibrations, respectively. The doublet at 1459 cm^−1^ is associated with N-CH_3_ asymmetric deformation. Finally, the bands at 1596 and 1707 cm^−1^ are attributed to ν(C=N) and ν(C=O) stretching vibrations, respectively [53]. The FT-IR spectrum of methomyl shows six characteristic contributions (Figure 5B). The intense bands at 670 and 1715 cm^−1^ are associated with the S-CH_3_ and C=O stretching vibrations [54,55], which were also detected in the Raman spectrum at 667 and 1707 cm^−1^, respectively. Bands at 1504 and 3304 cm^−1^ are attributed to the C-H bending vibration of the CH_3_ group and the N-H stretching vibration, respectively.

On the other hand, benomyl exhibits well-defined Raman bands across almost the entire range, with bands at 402, 620, and 725 cm^−1^ corresponding to C-O rocking, N-C-N bending, and out-of-plane C-H bending vibrations, respectively (Figure 5A). Rocking vibrations of the CH_2_ groups are evident at 782 and 844 cm^−1^. Two medium-intensity bands are observed at 962 and 1020 cm^−1^, associated with C-H bending and C-N rocking vibrations, respectively. The most intense band for benomyl appears at 1275 cm^−1^, corresponding to the in-plane bending vibration of C-C-H. The bands at 1362 and 1477 cm^−1^ are attributed to CH_2_ wagging and C-N-H rocking vibrations. Finally, the bands at 1606 and 1725 cm^−1^ are assigned to C=C and C=O stretching vibrations, respectively [56]. Some of these contributions are also observed in the FT-IR spectrum, with bands at 730, 1452, and 1640 cm^−1^ (Figure 5B). Additionally, two bands at 2932 and 3323 cm^−1^ related to the C-H stretching of the CH_3_ group and N-H stretching vibrations [56], respectively, are observed. A summary of Raman and FT-IR contributions observed for carbamate pesticides is provided in Table 1.

### 3.2. Characterization of the Tomato Peel Sample

**Figure 6:**
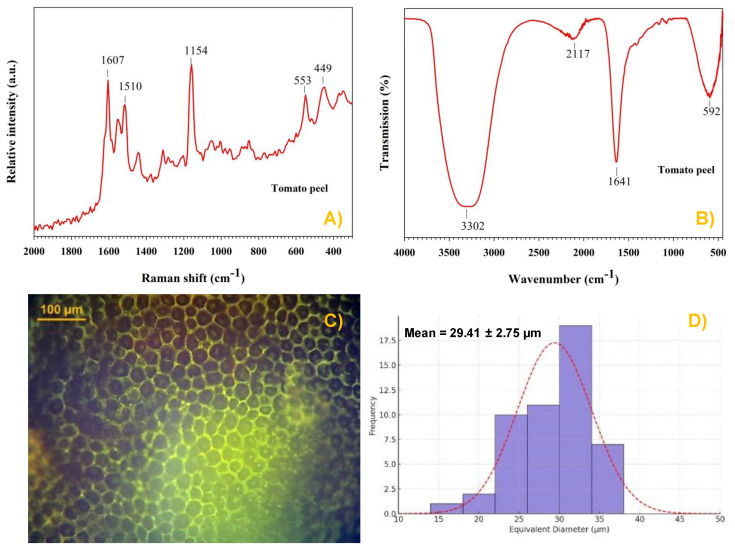
(**A**) Raman spectrum, (**B**) FT-IR spectrum, (**C**) optical microscopy image, and (**D**) distribution profile and average polygonal width of tomato peel.

Tomato peel contains various compounds, including amino acids, phenolic compounds, pectin, and carotenoids, featuring functional groups such as -COOH, -OH, and -NH_2_ [57]. The Raman spectrum of tomato peel is shown in Figure 6A. Three prominent bands are observed: the first, at 1607 cm^−1^, is associated with the aromatic ring of phenolic groups; two other prominent bands, at 1154 and 1510 cm^−1^, correspond to the ν(C-C) and ν(C=C) stretching vibrations of carotenoid compounds, respectively [58]. These Raman bands serve as distinctive markers for identifying carotenoids in tomatoes, offering valuable insights into their chemical composition and quality [59]. Additionally, two smaller bands at 449 and 553 cm^−1^, related to the presence of lycopene, are also detected [58].

The FT-IR spectrum of tomato peel shows four characteristic bands at 592, 1641, 2117, and 3302 cm^−1^, Figure 6B. The broad band with a maximum at 3302 cm^−1^ is attributed to O-H stretching vibration of phenolic and carboxylic groups. The band at 2117 cm^−1^ is associated with C-H stretching vibrations, while the band at 1641 cm^−1^ is attributed to the stretching of C=C bonds [57,60]. The band at 592 cm^−1^ may correspond to bending modes of functional groups present in the complex matrix of the peel. On the fresh tomato peel surface, clearly defined cell wall contours can be observed (Figure 6C). Moreover, the tomato peel exhibits a well-organized, highly compact, and interconnected polygonal structure, characterized by concave central regions and elevated contour edges above the epidermal level, with polygonal widths of approximately 29.41 ± 2.75 μm (Figure 6D).

### 3.3. Analysis of the Pesticides in Tomato Peel Samples

**Figure 7:**
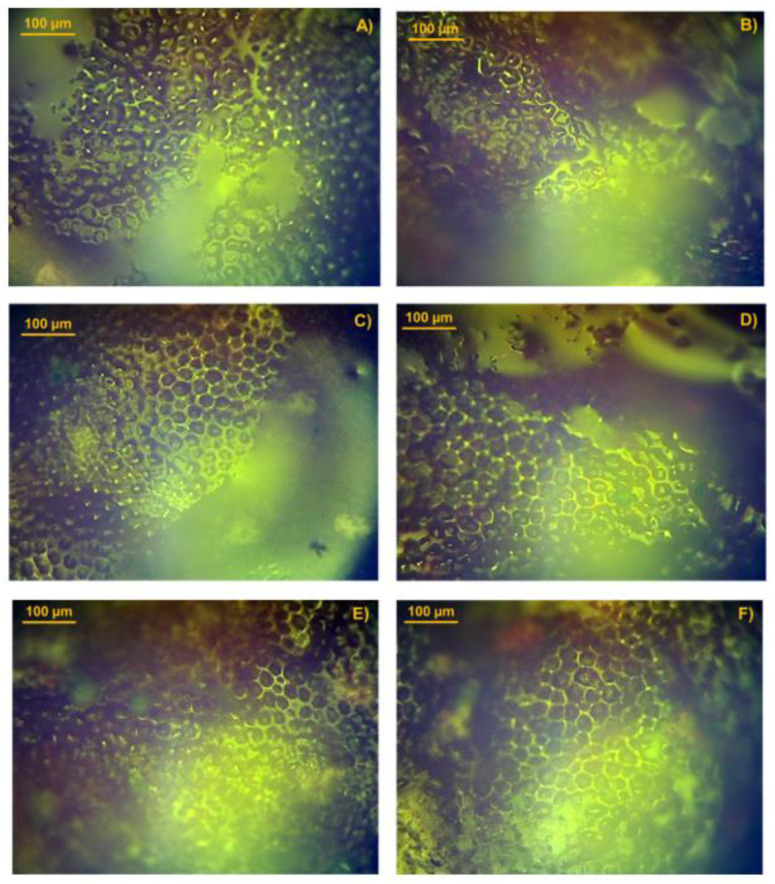
Optical microscopy images of tomato peel samples after treatment with: (**A**) dichlorvos, (**B**) methamidophos, (**C**) lambda-cyhalothrin, (**D**) cypermethrin, (**E**) methomyl, and (**F**) benomyl, pesticides.

After the application of pesticides, tomato peels were analyzed by optical microscopy, as shown in Figure 7. Compared to the untreated sample (Figure 6C), noticeable changes on the surface can be observed. In the samples treated with organophosphorus pesticides, liquid residues are observed within the polygon structures on both surfaces (Figure 7A,B). Samples treated with pyrethroids exhibit a blurred overall shape and contour (Figure 7C,D), probably due to the viscosity of the lambda-cyhalothrin and cypermethrin pesticides, Finally, in the samples treated with methomyl and benomyl, a small amount of solid residue can be observed, resulting from solvent evaporation (Figure 7E,F). Therefore, no evidence of cell wall disruption caused by pesticide application was observed.

#### 3.3.1. Organophosphorus Pesticides in Tomato Peel Samples

**Figure 8:**
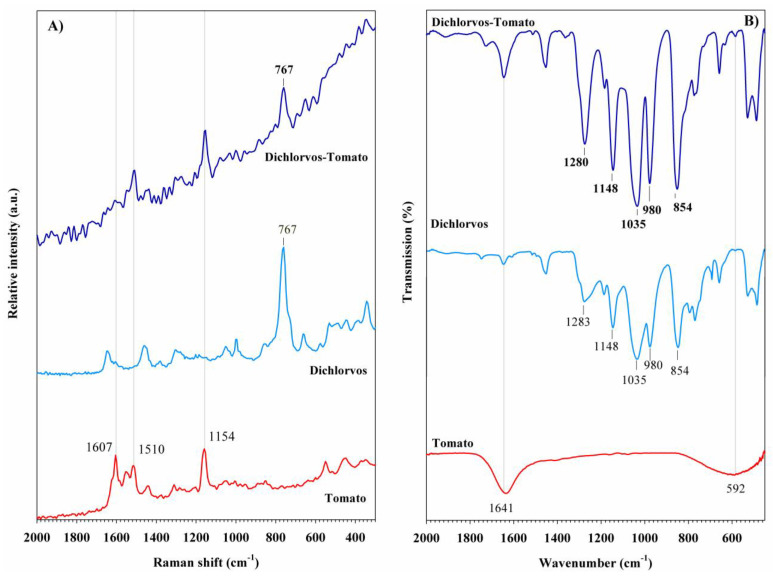
(**A**) Raman spectra and (**B**) FT-IR spectra of dichlorvos pesticide detected in tomato peel samples.

After applying organophosphorus pesticides to tomato peels, the samples were characterized using spectroscopic techniques. To identify the pesticides, the Raman and FT-IR spectra of treated samples were compared with those of untreated tomato peel. The Raman and FT-IR spectra of tomato peel treated with dichlorvos are shown in Figure 8. The characteristic Raman bands of carotenoid compounds in the peel samples appear at 1607, 1510, and 1154 cm^−1^, even after the application of both pesticides (Figure 8A). In the sample treated with dichlorvos, a Raman band at 767 cm^−1^, associated with the ν(P-O) stretching vibration, is observed. The presence of this band confirms the detection of dichlorvos in the tomato peel. This result is consistent with the Raman spectrum of dichlorvos reagent, in which the same band exhibits the highest intensity across the spectral range. In the FT-IR analysis, the bands at 1641 and 592 cm^−1^, originating from the tomato peel, remain evident after pesticide application (Figure 8B). Additionally, the FT-IR spectrum of the tomato peel treated with dichlorvos shows five intense bands at 854, 980, 1035, 1148, and 1280 cm^−1^, further supporting the presence of the pesticide.

**Figure 9:**
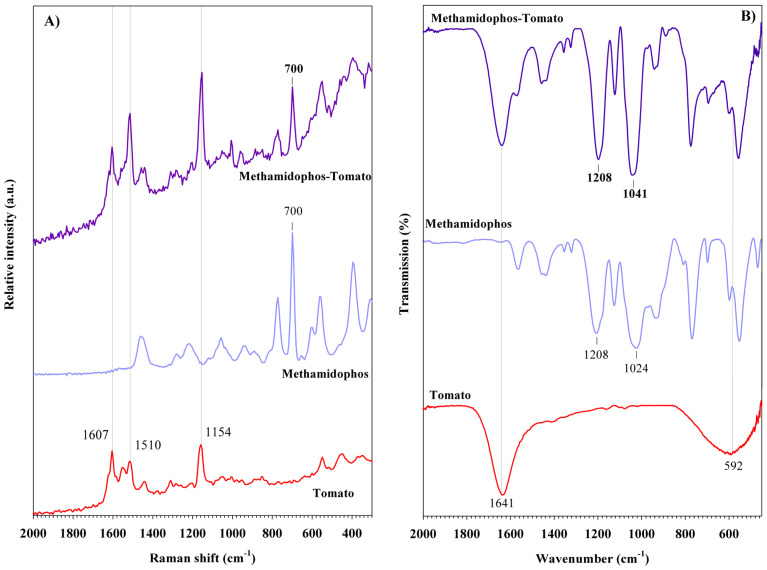
(**A**) Raman spectra and (**B**) FT-IR spectra of methamidophos pesticide detected in tomato peel samples.

For methamidophos, a Raman band at 700 cm^−1^, attributed to the ν(C-S) vibration, is detected (Figure 9A). The FT-IR spectrum confirms the presence of methamidophos through two bands at 1041 and 1208 cm^−1^, which correspond to the r(CH_3_) and ν(PO_2_) vibrations (Figure 9B), respectively.

#### 3.3.2. Pyrethroid Pesticides in Tomato Peel Samples

**Figure 10:**
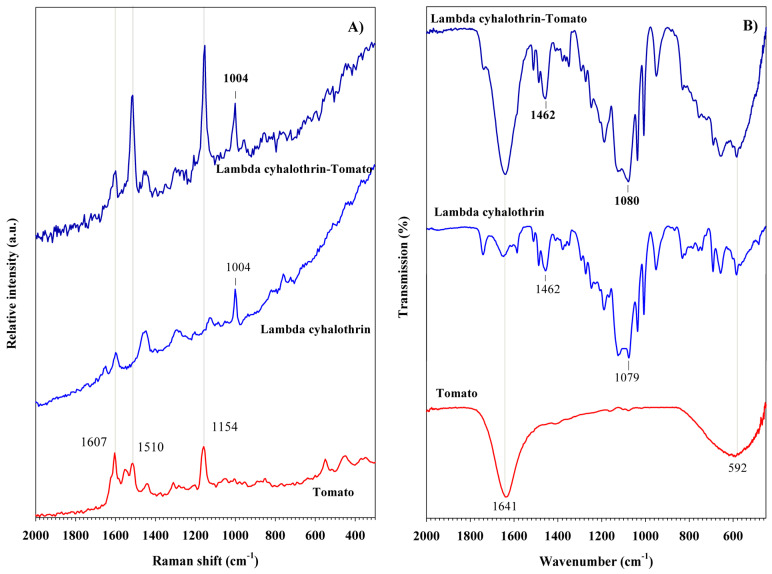
(**A**) Raman spectra and (**B**) FT-IR spectra of lambda cyhalothrin pesticide detected in tomato peel samples.

The Raman and FT-IR spectra of tomato peel samples treated with lambda-cyhalothrin are shown in Figure 10. These spectra exhibit well-defined and intense bands characteristic of the respective compounds, indicating the presence of successful pesticide residues. In the Raman spectrum of the sample treated with lambda-cyhalothrin (Figure 10A), a band at 1004 cm^−1^, corresponding to vibrational modes of the benzene ring, is detected. Complementary FT-IR analysis (Figure 10B) supports the presence of this pesticide, showing two characteristic bands at 1080 and 1462 cm^−1^, which are attributed to C-O and C=C stretching vibrations, respectively.

**Figure 11:**
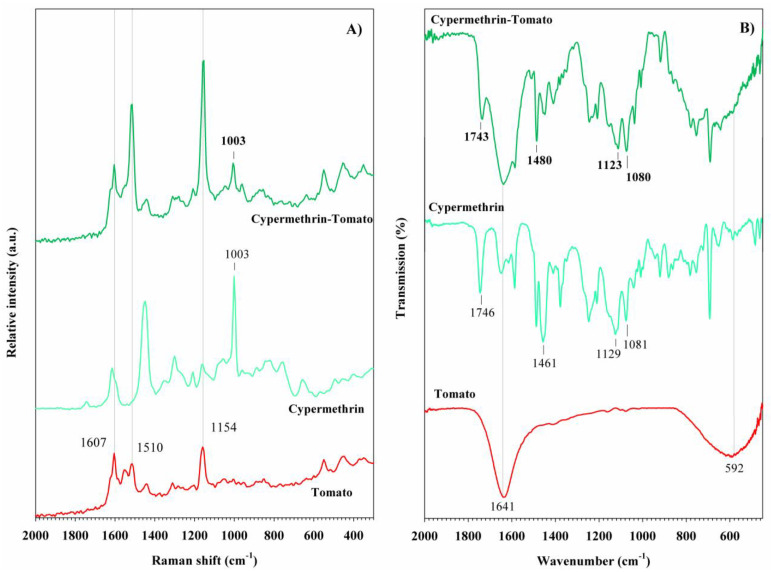
(**A**) Raman spectra and (**B**) FT-IR spectra of cypermethrin pesticide detected in tomato peel samples.

In the case of the sample treated with cypermethrin (Figure 11A), the benzene ring breathing mode is observed at 1003 cm^−1^. This band is particularly useful for identifying cypermethrin, as it shows high intensity in the Raman spectrum of the cypermethrin reagent. Furthermore, the FT-IR spectrum shows two bands associated with ν(C-O) stretching vibrations at 1080 and 1123 cm^−1^, along with two additional bands corresponding to ν(C=C) and ν(C=O) stretching at 1480 and 1743 cm^−1^, respectively, confirming the detection of cypermethrin on the tomato peel (Figure 11B).

#### 3.3.3. Carbamate Pesticides in Tomato Peel Samples

**Figure 12:**
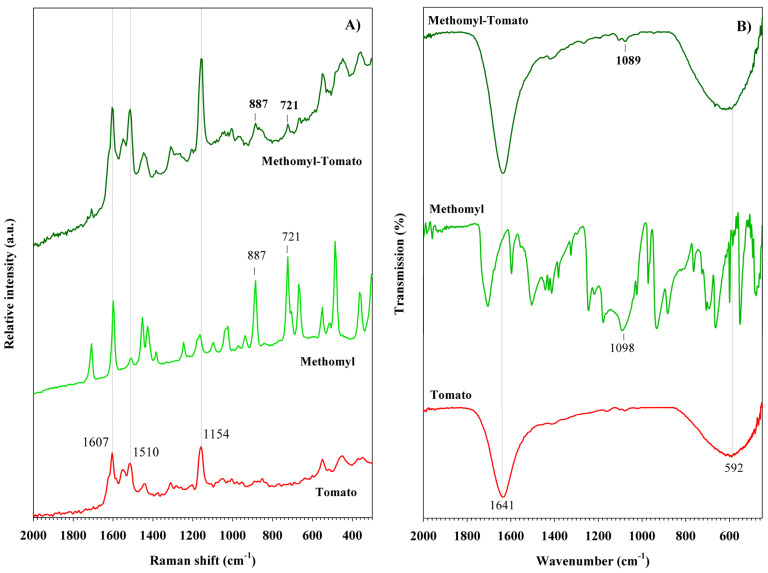
(**A**) Raman spectra and (**B**) FT-IR spectra of methomyl pesticide detected in tomato peel samples.

**Figure 13:**
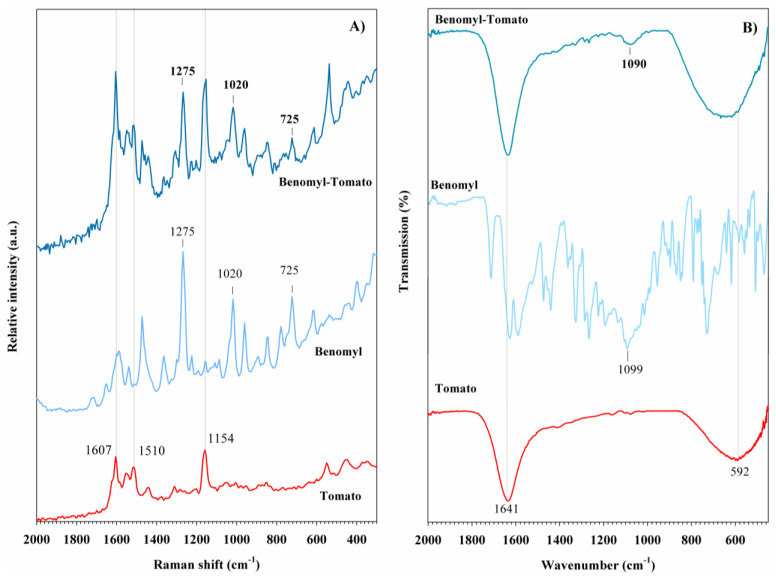
(**A**) Raman spectra and (**B**) FT-IR spectra of benomyl pesticide detected in tomato peel samples.

Compared to the Raman and FT-IR spectra of methomyl and benomyl reagents, the tomato samples treated with these pesticides exhibit lower spectral complexity (Figure 12 and Figure 13). In the Raman spectrum of the sample treated with methomyl, the characteristic carotenoid bands are clearly observed (Figure 12A). The band at 887 cm^−1^ corresponds to the ν(C-N) vibration, while the band at 721 cm^−1^ is attributed to the ν(S-CH_3_) stretching vibration. In the Raman spectrum of tomato treated with benomyl (Figure 13A), three bands at 725, 1020, 1275 cm^−1^, corresponding to the pesticide, are observed. The assignments for these bands are summarized in Table 1. In the FT-IR spectra of carbamate pesticides, the presence of methomyl and benomyl is indicated only by very weak contributions at 1089 and 1090 cm^−1^, respectively (Figure 12B and Figure 13B). These are both associated with ν(C-N) stretching vibrations.

### 3.4. Phytotoxicity of Tomato Under Pesticides Presence

Pesticides are essential during the critical growth phases of tomato crops to mitigate weed competition and pest-related damage. However, frequent pesticide application may cause phytotoxicity, which is manifested as plant damage, including impaired germination, growth, and yield [61,62].

**Figure 14:**
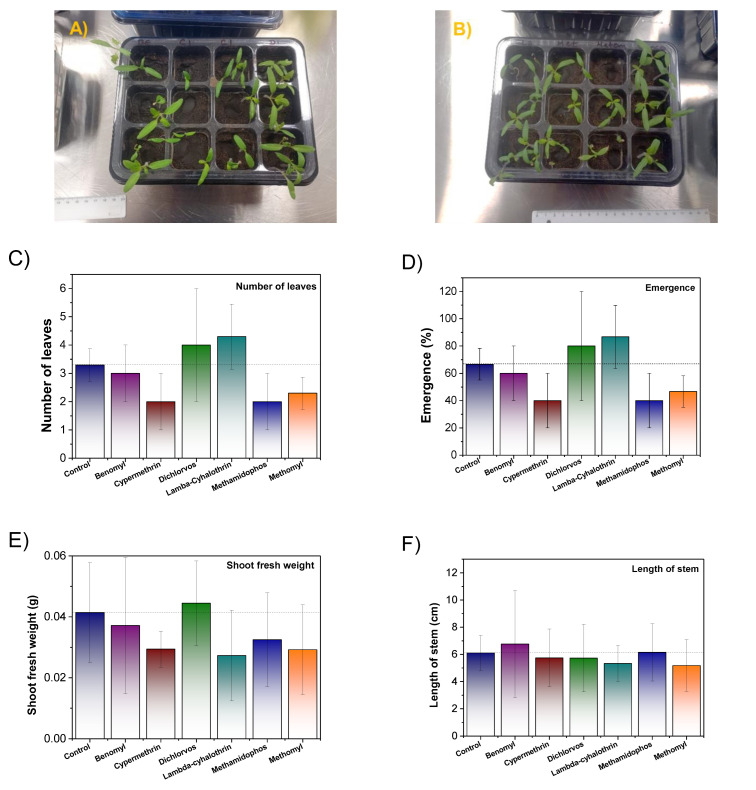

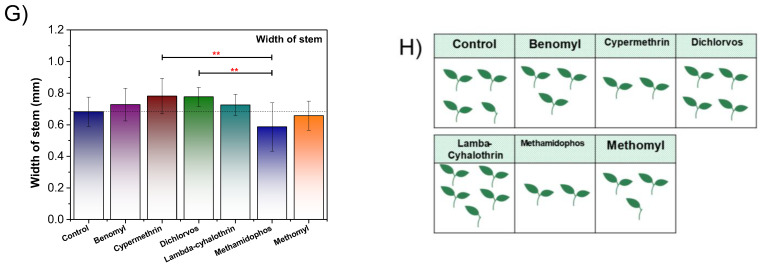
(**A**,**B**) Evidence of tomato seed growth and results from phytotoxicity of (**C**) number of emerged leaves, (**D**) emergence %, (**E**) shoot fresh weight, (**F**) length of stem, (**G**) width of stem, and (**H**) representative growth of tomato seeds in the presence of pesticides: dichlorvos; methamidophos; lambda cyhalothrin; cypermethrin; methomyl; benomyl, according to the number of leaves.

Phytotoxicity was assessed through visual analysis of the tomato plants germinated in the presence of the different pesticides to evaluate the specific effects of each compound. Figure 14A,B display evidence of grown tomato plants, whereas Figure 14C,D display the number of emerged leaves and the emergence percentage after the evaluation period. Germination for the control was positive, with 3.3 ± 0.57 leaves per spot, at a 66.6% success rate. The control had no deformations from phytotoxic effects. Compared to other works, the germination percentage is lower than, for example, from Loera-Serna et al. [63] in their effort of analyzing germination in presence of metal–organic materials, who reported 90% when employing tomatoes. Similarly, other works, such as that of Sierra Montes et al. [64], demonstrated an 8.5 ± 0.6 number of plants emerged; however, it is not clear how many seeds were employed. These authors report no significant differences in the analysis. The emergence percentage varied depending on the pesticide used (Figure 14D). The treatments with dichlorvos and lambda cyhalothrin resulted in values of 4.00 ± 2.00 (80%) and 4.33 ± 1.15 (86.6%), respectively. In contrast, benomyl, cypermethrin, methamidophos, and methomyl resulted in lower emergence percentages, i.e., 3.00 ± 1.00 (60%), 2.00 ± 1.00 (40%), 2.00 ± 1.00 (40%), and 2.33 ± 0.57 (46.66%). From these results, the sample with the highest standard deviation was dichlorvos based on the number of leaves emerged and emergence %. Despite the reduced emergence percentages, no negative effects on morphology or growth were observed across treatments. This can be observed in the shoot fresh weigh measurements, as shown in Figure 14E, which were similar for all samples in the presence of pesticides. The control displays a value of 0.041 ± 0.016, while the samples with pesticides exhibit the following values: benomyl 0.037 ± 0.022, cypermethrin 0.029 ± 0.005, dichlorvos 0.044 ± 0.013, lambda-cyhalothrin 0.027 ± 0.015, methamidophos 0.032 ± 0.015, and methomyl 0.029 ± 0.014. The whole samples display similar standard deviations in shoot fresh weight, with the exception of dichlorvos.

In the case of stem length, the sample treated with benomyl showed the highest value (6.757 ± 3.924), slightly exceeding the control (6.1 ± 1.288), as shown in Figure 14F. This sample displays the highest standard deviation. The samples treated with cypermethrin, dichlorvos, lambda-cyhalothrin, methamidophos, and methomyl showed stem lengths comparable to the control, with values of 5.737 ± 2.123, 5.725 ± 2.480, 5.325 ± 1.325, 6.150 ± 2.106, and 5.175 ± 1.908, respectively. Stem width was also analyzed (Figure 14G). The sample treated with cypermethrin had the greatest stem width (0.781 ± 0.112), which was slightly above that of the control (0.682 ± 0.092). Other notable values were observed for samples treated with benomyl (0.727 ± 0.103), dichlorvos (0.776 ± 0.060), and lambda-cyhalothrin (0.725 ± 0.061). Finally, the lowest values were observed for samples treated with methamidophos (0.586 ± 0.153) and methomyl (0.657 ± 0.092).

In other types of study involving cowpea cultivation, it is of note that cypermethrin was reported to induce a delay in seed germination as well as growth. However, this feature was not observed in this case [65]. For dichlorvos, other studies in cowpea seeds have shown a germination of 92% (*p* < 0.05) [66]. Dichlorvos has shown germination % c.a. 40–80% after 312 h, whereas this was between 60 and 90% for tomato seeds exposed to lambda-cyhalothrin, which reduced as the dose increased [67]. The literature studying methamidophos is not clear in this topic. Finally, methomyl has shown negative effects on the growth of leaves and shoots of tomatoes cultivated in Saudi Arabia [68].

For the phytotoxicity analysis, a one-way ANOVA was performed, followed by Tukey’s post hoc test. No statistically significant differences were observed in the number of emerged leaves (*p* = 0.122), emergence % (*p* = 0.122), shoot fresh weight (*p* = 0.206), or length of stem (*p* = 0.943), based on the established significance level (*p* < 0.05). However, a significant difference was observed for the width of stem (*p* = 0.0041). Tukey’s multiple comparisons test revealed that the treatment with methamidophos significantly reduced the width of the stem compared to cypermethrin (*p* = 0.0062, **) and dichlorvos (*p* = 0.0083, **), suggesting that methamidophos negatively affects stem thickness.

## 4. Discussion

Organophosphorus, pyrethroid, and carbamate compounds have become some of the most widely used chemicals for controlling agricultural pests since the ban on organochlorine pesticides. Organophosphorus pesticides are chemical derivatives of acids such as phosphoric, phosphonic, or phosphorothioic acids [69]. These compounds represent the most widely used class of pesticides [70]. The primary acute toxic effect of exposure to these substances is the inhibition of acetylcholinesterase (AChE) activity, which is essential for the proper functioning of the central nervous system (CNS) [71]. Common organophosphorus pesticides include malathion, diazinon, parathion, and chlorpyrifos [72]. In developing countries, dichlorvos is among the most commonly used organophosphate pesticides [73,74]. The World Health Organization (WHO) classifies both dichlorvos and methamidophos as Class 1b pesticides, indicating that they are highly hazardous chemicals [75], as shown in Table 2. Dichlorvos is commonly used in both domestic and agricultural settings to control pests [73]. It is applied in agricultural, industrial, commercial, and household environments to control flies, spider mites, and caterpillars, and is used on food crops such as mushrooms, lettuce, and tomatoes [76]. Methamidophos is a systemic insecticide effective against chewing and sucking insects [77]. Due to its high efficiency, it is widely applied to crops such as potatoes, beans, citrus, sugar beet, rice, cotton, melon, and tomatoes, despite being restricted in many countries [78,79]. Dichlorvos and methamidophos are both acutely and chronically toxic, with oral LD_50_ (lethal dose) values of 57–108 mg/Kg and 30 mg/Kg, respectively.

Pyrethroids are synthetic chemical analogues of pyrethrins. Their toxicity has been linked to endocrine system disruption, potentially affecting hormonal balance and reproductive health [80]. Common pyrethroids include permethrin, deltamethrin, and cypermethrin [81]. The two pyrethroids analyzed in this study, lambda cyhalothrin and cypermethrin, are classified by the WHO as Class II (moderately hazardous) pesticides, with LD_50_ values of 56 and 250 mg/Kg, respectively. These compounds are Type II pyrethroids, characterized by the presence of a cyano group in the α-position [82], as shown in Figure 2. Lambda cyhalothrin is an insecticide with insecticidal and acaricidal properties. It acts rapidly and is considered one of the most potent pyrethroids in global use [83]. It is used to protect fruits and vegetables (e.g., strawberries, lettuce, and tomatoes), as well as cereals and cotton. Its low solubility in water contributes to its long residual activity [84]. Cypermethrin is the most widely used pyrethroid worldwide and is frequently detected in vegetables and fruits. It is applied across nearly all types of agricultural systems [85]. The maximum residue limits (MRL) established for these pyrethroids in tomatoes are 0.3 mg/Kg for lambda-cyhalothrin and 0.2 mg/Kg for cypermethrin, respectively.

Carbamates are derivatives of carbamic acid and are widely employed as insecticides, herbicides, and fungicides [86]. Similarly to organophosphorus compounds, carbamate pesticides act by inhibiting AChE activity. They are used in countries where agriculture is a major industry and effective pest management is essential [87]. Consequently, carbamate residues are frequently found in fruits and vegetables [88]. Common carbamate pesticides include carbaryl, carbofuran, aldicarb, and methomyl [89]. Methomyl, an oxime carbamate, is effective against a range of pests, including spiders, aphids, beetles, flies, moths, and ticks [90]. It is highly toxic, exhibiting an LD_50_ value of 17 mg/Kg, the lowest among the pesticides listed in Table 2. Methomyl has been shown to induce genotoxic effects, including sister chromatid exchanges, chromosomal alterations, and the formation of micronucleus [91]. Although it is used on several crops such as corn, potatoes, and soybeans, methomyl is primarily applied to tomato crops [92,93,94]. Benomyl, a broad-spectrum benzimidazole fungicide, is also widely used in agriculture [95,96]. Residues of benzimidazole compounds, including benomyl, have been detected in tomato samples [32,56,97]. Benomyl is known to induce chromosomal changes and to disrupt the microtubule cytoskeleton in liver cells [98]. The MRLs for carbamate pesticides in tomatoes range from 1 to 5 mg/Kg.

**Table 2:** Classification of the pesticides analyzed in this study [99,100,101,102,103].

Pesticide	Type of Pesticide	Group	CAS Number	WHO Class ^a^	LD_50_ (mg/Kg)	MRL ^b^ in Tomato (mg/L)
Dichlorvos	Organophosphorus	Insecticide	62-73-7	Ib	57–108	0.1–0.5
Methamidophos	Organophosphorus	Acaricide, insecticide	10265-92-6	Ib	30	2
Lambda cyhalothrin	Pyrethroid	Insecticide	91465-08-6	II	56	0.3
Cypermethrin	Pyrethroid	Acaricide, insecticide	52315-07-8	II	250	0.2
Methomyl	Carbamate	Insecticide	16752-77-5	Ib	17	1
Benomyl	Carbamate	Fungicide	17804-35-2	U	>10,000	2.5–5

^a^ WHO Toxicity classes: Class II, moderately hazardous; Class Ib, highly hazardous; U, unlikely to present acute hazard in normal use. ^b^ Maximum Residue Limit as established by WHO.

Among spectroscopic methods, the detection of pesticides in tomato samples is commonly performed using SERS [22,29,37], which often requires specific sample pretreatments or the incorporation of nanomaterials. The results obtained in this work demonstrate the successful identification of organophosphorus, pyrethroid, and carbamate pesticides in tomato peel samples using conventional Raman and Fourier Transform Infrared (FT-IR) spectroscopy. In addition to its portability, Raman spectroscopy proved remarkably effective, providing well-defined and intense bands that enabled the detection of each pesticide based on its characteristic vibrational modes. For organophosphorus compounds, Raman bands corresponding to the ν(P-O) and ν(C-S) stretching vibrations at 767 and 700 cm^−1^ were especially useful for identifying dichlorvos and methamidophos, respectively. In the case of pyrethroids, benzene ring breathing modes observed at 1004 and 1003 cm^−1^ facilitated the detection of lambda-cyhalothrin and cypermethrin, respectively. For carbamates, bands related to C-N vibrations at 887 and 1020 cm^−1^ supported the identification of methomyl and benomyl. These findings are consistent with previous studies using SERS, in which similar bands were employed for quantitative analysis in fruits, vegetables, and other matrices. For instance, bands at 700, 1000, 1002, 674, and 725 cm^−1^ have been used for the quantification of methamidophos, lambda-cyhalothrin, cypermethrin, methomyl, and benomyl, respectively [43,104,105,106].

Although FT-IR spectroscopy is less suitable for in-field analysis compared to Raman spectroscopy, it serves as a valuable complementary technique for confirming the presence of pesticides. For organophosphorus compounds, FT-IR bands at 1280 and 1208 cm^−1^, associated with P-O vibrations, supported the detection of dichlorvos and methamidophos. In pyrethroid-treated samples, ν(C=C) stretching vibrations were observed at 1462 cm^−1^ for lambda-cyhalothrin and 1480 cm^−1^ for cypermethrin. For carbamates, weak but identifiable bands at 1089 and 1090 cm^−1^, corresponding to ν(C-N) vibrations, confirmed the presence of methomyl and benomyl.

Regarding the phytotoxicity test, the results indicate that the presence of pesticides can influence tomato seed germination and early growth (Figure 14H). While some pesticides, such as dichlorvos and lambda-cyhalothrin, maintained relatively high emergence percentages, others, including benomyl, cypermethrin, methamidophos, and methomyl, significantly reduced them (Figure 14D). Despite these variations, no visible morphological damage was observed, and the shoot fresh weight and length remained largely comparable to those of the control. These results suggest that although certain pesticides may reduce seed emergence, their impact on subsequent plant growth is minimal under the tested conditions. Future studies involving biochemistry analysis and different concentrations to evaluate the effect on phytotoxicity are recommended.

## 5. Conclusions

This study shows that organophosphorus, pyrethroid, and carbamate pesticides can be detected in tomato peel samples using spectroscopic analysis without the need for sample pretreatment. In particular, Raman spectroscopy offers advantages for in situ monitoring due to its portability, non-destructive nature, and high molecular specificity. The ability to detect key functional groups, such as P-O, C-S, C=C, and C-N bonds, demonstrates the potential of molecular vibrational fingerprinting for the selective identification of pesticides, even in complex biological matrices. The detection of characteristic vibrational bands without the need for sample pretreatment highlights the strong potential of this technique for rapid, routine monitoring in quality control and food safety applications.

## Figures and Tables

**Table 1:** Raman and FT-IR vibrational mode assignments of organophosphorus, pyrethroid, and carbamate pesticides.

	Raman		FT-IR	
Pesticide	Raman Shift (cm^−1^)	Assignment	Wavenumber (cm^−1^)	Assignment
**Dichlorvos**			656	ν(C-Cl)
	**767**	ν(P-O)	768	ν_s_(P-O-C)
	852	ν_IP_(P-O-C)	**854**	ν_IP_(P-O-C)
	995	ν_OOP_(P-O-C)	**980**	ν(P-O-C)
			**1035**	ν_as_(P-O-C)
			**1148**	ν(C-O)
	1309	ν(P=O)	**1283**	ν(P=O)
	1467	β(CH_3_)	1457	β(CH_3_)
	1649	ν(C=C)	1651	ν(C=C)
			2859–2958	ν(C-H) of vinyl group
**Methamidophos**	398	τ(N-H)		
	563	ω(N-H)		
	**700**	ν(C-S)		
	776	ν(P-O), ω(N-H)	768	ν(P-O)
	941	ν(C-O) + ν(P-O)	937	ν(C-O) + ν(P-O)
	1060	r_ip_(CH_3_)	**1024**	r_ip_(CH_3_)
	1222	ν(PO_2_)	**1208**	ν(PO_2_)
	1450	β(CH_3_)	1437	β(CH_3_)
			1568	δ(NH_2_)
			2951, 3106	ν(CH_3_)
			3237	ν(NH_2_)
**Lambda-cyhalothrin**	764	γ_ip_ of benzene ring		
	**1004**	Breathing of benzene ring		
	1294	ν(C-O)	**1079**	ν(C-O)
	1459	δ(C-H)		
			**1462**	ν(C=C)
	1596	ν of benzene ring	1590	ν of benzene ring
			1747	ν(C=O)
			2870–2965	ν(C-H)
			3423	ν(O-H)
**Cypermethrin**	659	γ_ip_ of cyclopropyl		
	759	β_oop_(C-H) benzene	697, 784	β_oop_(C-H) benzene
	**1003**	Breathing of benzene ring		
			**1081, 1129**	ν(C-O)
	1166	δ(C-H) benzene		
	1308	ν_skeleton_ of benzene ring		
	1456	δ(C-H)		
	1618	ν(C=C)	**1461**	ν(C=C)
	1737	ν(C=O)	**1746**	ν(C=O)
			2858, 2925, 2959	ν(C-H)
			3440	ν(O-H)
**Methomyl**	365	β(N-C_2_)		
	487	r(C=O)		
	667, **721**	ν(S-CH_3_)	670	ν(S-CH_3_)
	**887**	ν(C-N)		
			941	ν(C-O-C)
	1024	r(N-CH_3_)		
			**1098**	ν(C-N)
	1247	ν(N-C_2_)		
	1459	γ(N-CH_3_)		
	1596	ν(C=N)	1504	β(C-H) CH_3_
	1707	ν(C=O)	1715	ν(C=O)
			3304	ν(N-H)
**Benomyl**	402	r(C-O)		
	620	β(N-C-N)		
	**725**	β_oop_(C-H)	730	β(C-H)
	782	r(CH_2_)		
	844	r(CH_2_)		
	962	β(C-H)		
	**1020**	r(N-C)		
			**1099**	ν(C-N)
	**1275**	β_ip_(C-C-H)	1265	β_ip_(C-C-H)
	1362	ω(CH_2_)		
	1477	r(C-N-H)	1452	r(C-N-H)
	1606	ν(C=C)	1640	ν(C=C)
	1725	ν(C=O)		
			2932	ν (C-H) CH_3_
			3323	ν(N-H)

ν: stretching; ω: wagging; τ: twisting; β: bending; r: rocking; γ: deformation; δ: scissoring; ip: in plane; oop: out of plane; IP: in phase; OOP: out of phase; s: symmetric; as: asymmetric. *Bands identified in the tomato peel samples are shown in bold*.

## Data Availability

The original contributions presented in this study are included in the article. Further inquiries can be directed to the corresponding authors.

## Abbreviations

FT-IR	Fourier Transform Infrared
SERS	Surface-enhanced Raman spectroscopy
MRL	Maximum residue limit
